# Supplementary material for: Seroprevalence of SARS-CoV-2 antibodies in Saint Petersburg, Russia: a population-based study
Source: Sci Rep. 2021 Jun 21;11:12930. doi: 10.1038/s41598-021-92206-y (PMC8217236; doi:10.1038/s41598-021-92206-y)
Supplement: Supplementary file 1 — Supplementary information. [file 41598_2021_92206_MOESM1_ESM.pdf]

Supplementary materials

Title

Seroprevalence of SARS-CoV-2 antibodies in Saint Petersburg, Russia: a population-based study

Authors

Anton Barchuk, Dmitriy Skougarevskiy, Kirill Titaev, Daniil Shirokov, Yulia Raskina, Anastasia Novkunkskaya, Petr Talantov, Artur Isaev, Ekaterina Pomerantseva, Svetlana Zhikrivetskaya, Lubov Barabanova and Vadim Volkov

Statistical appendix

Let adult population of St. Petersburg be of size N, indexed with i, and characterised by a triplet $\langle Z, D, Y \rangle$. $Z_i = 1$ means that the i-th resident will be surveyed, with $Z_i = 0$ otherwise. Let $\mathbf{Z}$ be a vector of size N, its i-th element equals Z_i. In other words, $\mathbf{Z}$ indexes which city residents will be surveyed.

D marks the individual decision to participate in the survey when contacted: $D_i(\mathbf{Z}) = 1$: i-th individual volunteers to take part in the study, with $D_i(\mathbf{Z}) = 0$ otherwise. Let $\mathbf{D}$ be the volunteer vector of size N.

When participation is universal $D_i(\mathbf{Z}) = Z_i \forall i$. In reality one could observe non-zero refusal rates. In what follows we assume one-sided noncompliance.

Variable Y characterises antibody status (seroconversion) of the i-th individual and takes the following values: $\{1, 0\}$, where 1 — has antibodies to SARS-CoV-2 and 0 — does not have antibodies. Antibody status can be both observed and unobserved: $Y_i \equiv Y_i(\mathbf{Z}, \mathbf{D})$. We are able to observe antibody status for $Y_i(Z_i = 1, D_i = 1)$, i.e. for the surveyed individuals who agreed to volunteer in the study and were tested.

We are interested in population seroprevalence estimate $\pi \equiv 1/N \times \sum_{i=1}^{N} Y_i$. Having conducted our study we can estimate the naïve seroprevalence $\widehat{\pi}_{naive} \equiv 1/n \times \sum_{i=1}^{n} Y_i(Z_i = 1, D_i = 1)$, where n is the number of tested individuals. To arrive at population-level seroprevalence estimates the following assumptions are required:

Random sampling The survey is conducted such that $\Pr(\mathbf{Z} = \mathbf{c}) = \Pr\left(\mathbf{Z} = \mathbf{c}'\right)$ where $\mathbf{c}$ and $\mathbf{c}'$ are arbitrary survey vectors.

Stable unit treatment value assumption (SUTVA) Both observed and unobserved individual antibody status depends only on his/her antibody status and decision to participate in the survey and not on the decision of other study participants. The surveyed individuals maintain no contacts or other social interactions that could influence their individual decisions to participate in the study. Such interactions might arise in case of household sampling where all household members are invited to participate in the survey.

No unobservable characteristics of study participants Let $\mathbf{X} \in \mathbb{R}^{N \times M}$ be a matrix of observable individual characteristics. Its row $\mathbf{X}_i$ stores all characteristics of individual i that influence his/her decision to participate in the study when surveyed. Naïve seroprevalence estimate will be representative of the city population only when $\mathrm{cov}([\mathrm{D}|\mathrm{X}], [\mathrm{Z}|\mathrm{X}]) = 0$ and $\mathrm{cov}([\mathrm{D}|\mathrm{X}], [\mathrm{Y}|\mathrm{X}]) = 0$. That is, conditional on all observable characteristics of individuals, the decision to participate in the study is orthogonal to both the conditional-on-observables survey inclusion and conditional-on-observables antibody status. While the former orthogonality assumption $[D|X] \perp [Z|X]$ is satisfied under random sampling of individuals, the latter is more problematic. First, no phone survey design could ensure full observability of all characteristics associated both with individual decision to volunteer and antibody status. Many variables might be left unobserved, rendering this orthogonality assumption unrealistic. Second, differing sets of observables can influence seroconversion and decision to volunteer. For instance, past travel history may be an important observable responsible for $D = 1$ while fully independent of actual antibody status $Y = 1$.

When the above assumptions are not satisfied the naïve seroprevalence estimate is biased. To account for this we rely on bivariate binary model with non-random selection in the spirit of Bärnighausen et al. (2011) [1]. First we assume that decision of a surveyed individual to agree to participate in the survey and come to the clinic test site is determined by a latent variable

$$D_i^* = \beta \boldsymbol{X}_i + \gamma T_i + \varepsilon_i \tag{1}$$

such that $D_i = 1$ if $D^* > 0$ and $D_i = 0$ otherwise. T_i is a variable equal to unity if surveyed individual was offered free taxi to and from the clinic test site during the phone survey, ε_i is the error term. We observe D only for n out of N individuals in the population.

We observe antibody status only for those with $D_i = 1$ and assume that seroconversion is determined by a latent variable

$$Y_i^* = \delta \boldsymbol{X}_i + \zeta_i \tag{2}$$

such that $Y_i = 1$ if $Y^* > 0$ and $Y_i = 0$ otherwise. ζ_i is the error term. Since we have offered taxi to random phone survey participants we can safely assume that $\mathrm{cov}([\mathrm{Y}|\mathrm{X}], \mathrm{T}) = 0$ and T becomes a valid exclusion restriction.

Table A1. Estimated error term correlation between selection stage and testing stage

**Design matrix X**	$\widehat{\rho}$**, CMIA**	$\widehat{\rho}$**, ELISA**
Demographic characteristics	-0.71 (-0.74;-0.68)	-0.71 (-0.74;-0.67)
Demographic and socioeconomic characteristics	-0.69 (-0.73;-0.67)	-0.69 (-0.72;-0.66)
Characteristics associated with seropositivity	0.32 (-1.0;1.0)	-0.94 (-1.0;-0.09)
Demographics, socioeconomic status and characteristics associated with seropositivity	0.50 (-1.0;1.0)	0.66 (-0.95;0.99)

We impose a structural assumption of independent and identically Normal-distributed error terms ε_i and ζ_i with nil mean and unit variance. Their joint cumulative distribution function is given by $\Phi(\varepsilon_i, \zeta_i, \rho)$ where ρ is covariance (correlation coefficient).

This bivariate probit is estimated with R package GJRM [2]. We use different definitions of seropositivity $Y = 1$ depending on antibody tests or combinations thereof and different set of variables in design matrix $\mathbf{X}$.

Our first step is to estimate $\widehat{\rho}$ and test whether it is statistically significantly different from zero. Estimates under different sets of variables are reported below with 95% CIs in parentheses are reported in Supplementary Appendix Table A1.

As our baseline we adopt a model where a rich set of demographic, socioeconomic, and seropositivity-related characteristics is included in the design matrix $\mathbf{X}$. In this model results from both the simulated CIs and Lagrange multiplier test with null $\rho = 0$ (not reported here, available at request) suggest that one cannot reject the null hypothesis of error term independence between the selection stage and antibody test result stage.

Under error term independence Heckman correction is not required to arrive at seroprevalence estimates for the entire city population when response is non-random. However, the naïve seroprevalence estimate can still be biased since the tested individuals are not representative of the city population. To circumvent this we use the estimated parameters from baseline seroconversion probit (see equation 2 above) and predict antibody status Y for all surveyed individuals regardless of their agreement to participate in the survey. Such (univariate) single imputation that assumes no unobserved confounders permits us to correct the naïve seroprevalence estimates for missing data for those individuals who have refused to get tested or did not visit the clinic.

Symmetric confidence intervals come from standard errors estimated with delta method. Results do not change qualitatively when we consider non-symmetric confidence intervals after Bayesian posterior simulation of the parameter vector estimate (not reported here, available at request).

Finally, $\widehat{\pi}$ using the formula

$$\widehat{\pi}_{corrected} = \frac{\widehat{\pi} + specificity - 1}{sensitivity + specificity - 1}; \;\; std.dev.\left(\widehat{\pi}_{corrected}\right) = \frac{std.dev.\left(\widehat{\pi}\right)}{sensitivity + specificity - 1}.$$

Statistical appendix references

1. Bärnighausen T, Bor J, Wandira-Kazibwe S, Canning D. Correcting HIV prevalence estimates for survey nonparticipation using Heckman-type selection models. Epidemiology. 2011;p. 27–35.
2. Marra G, Radice R. A joint regression modeling framework for analyzing bivariate binary data in R. Dependence Modeling. 2017;5(1):268–294.
3. Reiczigel J, Földi J, Ózsvári L. Exact confidence limits for prevalence of a disease with an imperfect diagnostic test. Epidemiology & Infection. 2010;138(11):1674–1678.

STROBE checklist for cross-sectional studies

	Item No	Recommendation	Page
Title and abstract			
	1a	Indicate the study's design with a commonly used term in the title or the abstract	See page 1
	1b	Provide in the abstract an informative and balanced summary of what was done and what was found	See page 1
Introduction			
Background/rationale	2	Explain the scientific background and rationale for the investigation being reported	See page 1
Objectives	3	State specific objectives, including any prespecified hypotheses	See page 1
Methods			
Study design	4	Present key elements of study design early in the paper	See page 2
Setting	5	Describe the setting, locations, and relevant dates, including periods of recruitment, exposure, follow-up, and data collection	See page 2
Participants	6	Give the eligibility criteria, and the sources and methods of selection of participants	See page 2
Variables	7	Clearly define all outcomes, exposures, predictors, potential confounders, and effect modifiers. Give diagnostic criteria, if applicable	See page 2
Data sources/ measurement	8	For each variable of interest, give sources of data and details of methods of assessment (measurement). Describe comparability of assessment methods if there is more than one group	See pages 2
Bias	9	Describe any efforts to address potential sources of bias	See page 2
Study size	10	Explain how the study size was arrived at	See page 2
Quantitative variables	11	Explain how quantitative variables were handled in the analyses. If applicable, describe which groupings were chosen and why	See page 2
Statistical methods	12a	Describe all statistical methods, including those used to control for confounding	See page 3
Statistical methods	12b	Describe any methods used to examine subgroups and interactions	See page 3
Statistical methods	12c	Explain how missing data were addressed	See page 3
Statistical methods	12d	If applicable, describe analytical methods taking account of sampling strategy	See page 2
Statistical methods	12e	Describe any sensitivity analyses	See page 3
Results			
Participants	13a	Report numbers of individuals at each stage of study – eg numbers potentially eligible, examined for eligibility, confirmed eligible, included in the study, completing follow-up, and analysed	See page 3
Participants	13b	Give reasons for non-participation at each stage	See page 3
Participants	13c	Consider use of a flow diagram	See page 3
Descriptive data	14a	Give characteristics of study participants (eg demographic, clinical, social) and information on exposures and potential confounders	See page 3
Descriptive data	14b	Indicate number of participants with missing data for each variable of interest	See page 3
Outcome data	15	Report numbers of outcome events or summary measures	See page 4
Main results	16a	Give unadjusted estimates and, if applicable, confounder-adjusted estimates and their precision (eg, 95% confidence interval). Make clear which confounders were adjusted for and why they were included	See page 4
Main results	16b	Report category boundaries when continuous variables were categorised	NA
Main results	16c	If relevant, consider translating estimates of relative risk into absolute risk for a meaningful time period	NA
Other analyses	17	Report other analyses done—eg analyses of subgroups and interactions, and sensitivity analyses	See page 5
Discussion			
Key results	18	Summarise key results with reference to study objectives	See page 5
Limitations	19	Discuss limitations of the study, taking into account sources of potential bias or imprecision. Discuss both direction and magnitude of any potential bias	See page 8
Interpretation	20	Give a cautious overall interpretation of results considering objectives, limitations, multiplicity of analyses, results from similar studies, and other relevant evidence	See page 5
Generalisability	21	Discuss the generalisability (external validity) of the study results	See page 6
Other information			
Funding	22	Give the source of funding and the role of the funders for the present study and, if applicable, for the original study on which the present article is based	See page 8

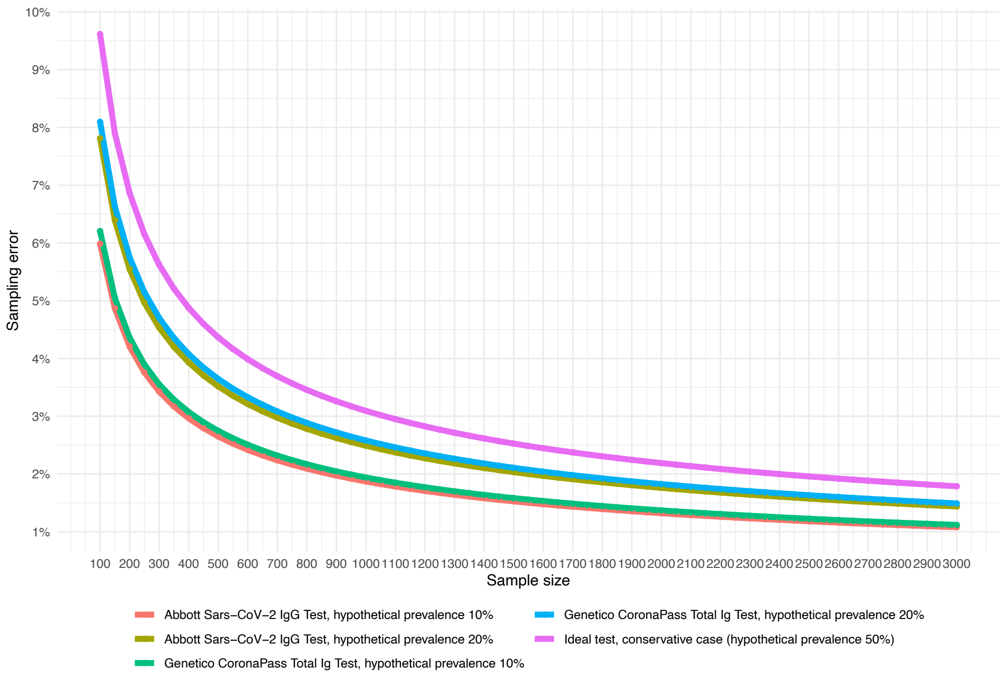

This chart reports the relationship between sampling error and sample size under different test characteristics and assumed hypothetical prevalence in the population. Calculations are made with the designated code [3].

Figure A1. Sampling error under different test characteristics

Table A2. Summary statistics of eligible phone survey respondents

		Overall	Not tested	Tested
Number of participants		6400	5362	1038
Age group				
	18–34	2333 (36.5)	1937 (36.1)	396 (38.2)
	35–49	2036 (31.8)	1679 (31.3)	357 (34.4)
	50–64	1271 (19.9)	1053 (19.6)	218 (21.0)
	65+	760 (11.9)	693 (12.9)	67 (6.5)
Sex				
	Female	3766 (58.8)	3114 (58.1)	652 (62.8)
	Male	2634 (41.2)	2248 (41.9)	386 (37.2)
Higher education				
	No	2108 (32.9)	1929 (36.0)	179 (17.2)
	Yes	4292 (67.1)	3433 (64.0)	859 (82.8)
Higher income				
	No	3484 (54.4)	2988 (55.7)	496 (47.8)
	Yes	2579 (40.3)	2076 (38.7)	503 (48.5)
	NA	337 (5.3)	298 (5.6)	39 (3.8)
Respondent lives alone				
	No	5206 (81.3)	4363 (81.4)	843 (81.2)
	Yes	1194 (18.7)	999 (18.6)	195 (18.8)
Respondent travelled abroad in the past 3 months				
	No	5982 (93.5)	5064 (94.4)	918 (88.4)
	Yes	418 (6.5)	298 (5.6)	120 (11.6)
Respondent started to wash hands more often				
	No	2190 (34.2)	1893 (35.3)	297 (28.6)
	Yes	4155 (64.9)	3419 (63.8)	736 (70.9)
	NA	55 (0.9)	50 (0.9)	5 (0.5)
History of illnesses in the past 3 months				
	No	4324 (67.6)	3747 (69.9)	577 (55.6)
	Yes	1997 (31.2)	1543 (28.8)	454 (43.7)
	NA	79 (1.2)	72 (1.3)	7 (0.7)
History of COVID 19 testing				
	No	5425 (84.8)	4621 (86.2)	804 (77.5)
	Yes	975 (15.2)	741 (13.8)	234 (22.5)
City district				
	Admiralteyskiy	265 (4.1)	205 (3.8)	60 (5.8)
	Frunzenskiy	477 (7.5)	403 (7.5)	74 (7.1)
	Kalininskiy	734 (11.5)	616 (11.5)	118 (11.4)
	Kirovskiy	437 (6.8)	384 (7.2)	53 (5.1)
	Krasnogvardeyskiy	534 (8.3)	457 (8.5)	77 (7.4)
	Moskovskiy	538 (8.4)	427 (8.0)	111 (10.7)
	Nevskiy	842 (13.2)	717 (13.4)	125 (12.0)
	Petrogradskiy	192 (3.0)	157 (2.9)	35 (3.4)
	Primorskiy	955 (14.9)	789 (14.7)	166 (16.0)
	Tsentralniy	342 (5.3)	290 (5.4)	52 (5.0)
	Vasileostrovskiy	250 (3.9)	207 (3.9)	43 (4.1)
	Vyborgskiy	834 (13.0)	710 (13.2)	124 (11.9)
Week of phone interview				
	May 18-24	473 (7.4)	372 (6.9)	101 (9.7)
	May 25-31	1333 (20.8)	1110 (20.7)	223 (21.5)
	June 1-7	1419 (22.2)	1190 (22.2)	229 (22.1)
	June 8-14	1496 (23.4)	1231 (23.0)	265 (25.5)
	June 15-21	981 (15.3)	817 (15.2)	164 (15.8)
	June 22-28	698 (10.9)	642 (12.0)	56 (5.4)
CMIA positive test				
	No	941 (14.7)	0 (0.0)	941 (90.7)
	Yes	97 (1.5)	0 (0.0)	97 (9.3)
	NA	5362 (83.8)	5362 (100.0)	0 (0.0)
ELISA positive test				
	No	928 (14.5)	0 (0.0)	928 (89.4)
	Yes	107 (1.7)	0 (0.0)	107 (10.3)
	NA	5365 (83.8)	5362 (100.0)	3 (0.3)
Smoking status *(paper survey)*				
	Never smoked	462 (7.2)	0 (0.0)	462 (44.5)
	Used to smoke	263 (4.1)	0 (0.0)	263 (25.3)
	Smoking now	195 (3.0)	0 (0.0)	195 (18.8)
	NA	5480 (85.6)	5362 (100.0)	118 (11.4)
Past history of allergies *(paper survey)*				
	No	631 (9.9)	0 (0.0)	631 (60.8)
	Yes	277 (4.3)	0 (0.0)	277 (26.7)
	NA	5492 (85.8)	5362 (100.0)	130 (12.5)
Chronic diseases or medication use *(paper survey)*				
	No	534 (8.3)	0 (0.0)	534 (51.4)
	Yes	418 (6.5)	0 (0.0)	418 (40.3)
	NA	5448 (85.1)	5362 (100.0)	86 (8.3)
Cold symptoms in the past 3 months *(paper survey)*				
	No	532 (8.3)	0 (0.0)	532 (51.3)
	Yes	418 (6.5)	0 (0.0)	418 (40.3)
	NA	5450 (85.2)	5362 (100.0)	88 (8.5)
Alcohol consumption frequency *(paper survey)*				
	Never	218 (3.4)	0 (0.0)	218 (21.0)
	Monthly	404 (6.3)	0 (0.0)	404 (38.9)
	Weekly or more often	318 (5.0)	0 (0.0)	318 (30.6)
	NA	5460 (85.3)	5362 (100.0)	98 (9.4)

Table A3. Sample means across study stages and in relation to a representative survey

	Called	Agreed	Tested	KOUZh-2018
N	6384	3370	1028	2977
N (paper-based survey)			911	
Male, %	41.3 (40.1-42.5)	37.4 (35.7-39)	37.1 (34.1-40)	40.2 (38.4-41.9)
Age, years	43.1 (42.7-43.5)	42.1 (41.6-42.6)	41.4 (40.6-42.3)	46.7 (46.1-47.3)
18-34, %	36.3 (35.1-37.4)	37.7 (36.1-39.4)	38.2 (35.3-41.2)	27.9 (26.3-29.5)
35-49, %	31.8 (30.7-33.0)	33 (31.4-34.6)	34 (31.1-36.9)	31.7 (30-33.3)
50-64, %	20.0 (19.0-20.9)	19.9 (18.6-21.3)	21.2 (18.7-23.7)	22.8 (21.3-24.3)
65+, %	12 (11.2-12.8)	9.3 (8.4-10.3)	6.5 (5.0-8.0)	17.6 (16.3-19.0)
Education				
Primary education, %	1.1 (0.8-1.4)	0.8 (0.0-0.6)	0.3 (0.5-1.1)	2.7 (2.1-3.3)
Complete secondary education, %	8.9 (8.2-9.6)	7.5 (6.6-8.4)	5.0 (3.6-6.3)	10.2 (9.1-11.3)
Special secondary education, %	23.2 (22.1-24.2)	20.1 (18.8-21.5)	11.9 (9.9-13.8)	39.5 (37.7-41.3)
Higher education, %	66.9 (65.7-68.0)	71.6 (70.1-73.1)	82.9 (80.6-85.2)	47.6 (45.8-49.4)
Employed, %	68.3 (67.1-69.4)	71.1 (69.6-72.7)	78.7 (76.2-81.2)	70.2 (68.6-71.8)
Self-reported health status				
Very good, %	19.9 (18.9-20.8)	16.9 (15.6-18.1)	15.2 (13-17.4)	7.8 (6.8-8.7)
Good, %	48.9 (47.6-50.1)	50.6 (48.9-52.3)	53.5 (50.4-56.6)	45.3 (43.5-47.1)
Satisfactory, %	28.5 (27.4-29.6)	29.8 (28.2-31.3)	29.7 (26.9-32.5)	39.2 (37.5-41)
Bad, %	2.4 (2.1-2.8)	2.4 (1.9-3.0)	1.6 (0.8-2.3)	7.1 (6.2-8.0)
Very bad, %	0.3 (0.2-0.5)	0.3 (0.1-0.5)	0.1 (0.0-0.3)	0.6 (0.3-0.9)
Lives alone, %	18.8 (17.8-19.7)	18.1 (16.8-19.4)	19 (16.6-21.4)	19.7 18.3-21.1)
Has cellphone, %	100.0 (100.0-100.0)	100.0 (100.0-100.0)	100 (100.0-100.0)	99.5 (99.3-99.8)
Self-reported smoking status (from paper-based survey)				
Never smoked, %			49.9 (46.7-53.2)	52.9 (51.1-54.7)
Smoking earlier, %			28.9 (25.9-31.8)	15.5 (14.2-16.8)
Smoking now, %			21.2 (18.5-23.8)	31.6 (30.0-33.3)
Consumes alcohol, %			76.6 (73.9-79.4)	74. (73.1-76.2)

95% confidence intervals in parentheses. "Called" means individuals who agreed to participate in the phone survey-"Agreed" are individuals who agreed to be contacted by the clinic to get tested-"Tested" are individuals who came to clinic and gave blood samples. KOUZh-2018 is the 2016 round of the Comprehensive monitoring of living conditions household survey carried out by the Federal State Statistics Service of Russia. We subset this survey to include only adults in St. Petersburg. We report only complete-case observations in terms of all variables, therefore the number of observations is slightly lower due to listwise deletion.

Table A4. SARS-CoV-2 seroprevalence estimates from bivariate probit models with different sets of individual characteristics for non-response bias correction and alternative definitions of seropositivity

Regressors included in bivariate probit model	CMIA or ELISA				CMIA and ELISA			
	Number of participants		Seroprevalence (95% CI)		Number of participants		Seroprevalence (95% CI)	
	Interviewed	Tested	Naïve	Single imputation	Interviewed	Tested	Naïve	Single imputation
Demographic characteristics	6400	1038	11.2% (9.3-13.2)	10.9% (9.0-12.8)	6397	1035	8.2% (6.5-9.9)	8.0% (6.3-9.6)
Demographic and socioeconomic characteristics	6063	999	11.6% (9.6-13.6)	11.5% (9.3-13.6)	6061	997	8.5% (6.7-10.2)	8.3% (6.4-10.2)
Characteristics associated with seropositivity	6267	1026	11.3% (9.3-13.2)	9.2% (7.5-10.9)	6264	1023	8.2% (6.5-10.0)	6.6% (5.0-8.1)
Demographics, socioeconomic status and characteristics associated with seropositivity	5953	990	11.6% (9.6-13.6)	9.7% (7.7-11.6)	5951	988	8.4% (6.7-10.2)	6.8% (5.2-8.5)

"Demographic characteristics" means the following variables: individual age group (18-34, 35-49, 50-64, 65+ years old) and sex. "Socioeconomic characteristics" means the following variables: higher education status and higher self-reported income level. "Characteristics associated with seropositivity" means the following variables: history of illness in the last 3 months, history of COVID-19 testing, whether respondent lives alone, change in hand washing habits during pandemic, week of the phone interview, and city district. All models include a variable indicating random offer of taxi transportation to and from the clinic test site for interviewed participants. All estimates are corrected for tests characteristics.

Table A5. SARS-CoV-2 seroprevalence estimates from bivariate probit models after re-weighting the sample with raking weights (age group and educational attainment level) estimated to match a 2016 representative survey of adult city residents

Regressors included in bivariate probit model	CMIA				ELISA			
	Number of participants		Seroprevalence (95% CI)		Number of participants		Seroprevalence (95% CI)	
	Interviewed	Tested	Naïve	Single imputation	Interviewed	Tested	Naïve	Single imputation
Demographic characteristics	6400	1038	9.0% (7.2-10.8)	8.5% (6.7-10.4)	6397	1035	10.5% (8.6-12.3)	9.6% (7.8-11.4)
Demographic and socioeconomic characteristics	6063	999	9.3% (7.5-11.1)	8.9% (6.4-11.3)	6061	997	10.8% (8.9-12.8)	10.6% (7.9-13.2)
Characteristics associated with seropositivity	6267	1026	9.0% (7.2-10.8)	6.7% (5.2-8.2)	6264	1023	10.5% (8.6-12.4)	8.2% (6.6-9.9)
Demographics, socioeconomic status and characteristics associated with seropositivity	5953	990	9.3% (7.5-11.2)	7.2% (5.1-9.3)	5951	988	10.8% (8.9-12.8)	8.8% (6.5-11.0)

"Demographic characteristics" means the following variables: individual age group (18-34, 35-49, 50-64, 65+ years old) and sex. "Socioeconomic characteristics" means the following variables: higher education status and higher self-reported income level. "Characteristics associated with seropositivity" means the following variables: history of illness in the last 3 months, history of COVID-19 testing, whether respondent lives alone, change in hand washing habits during pandemic, week of the phone interview, and city district. All models include a variable indicating random offer of taxi transportation to and from the clinic test site for interviewed participants. All estimates are corrected for tests characteristics. Serosurvey sample was re-weighted with raking weights estimated to match the survey age group and educational attainment proportions in 2016 representative survey of adult city population (see Supplementary Appendix Table A3 for description of this survey and the target proportions). R package anesrake was used to compute the weights.

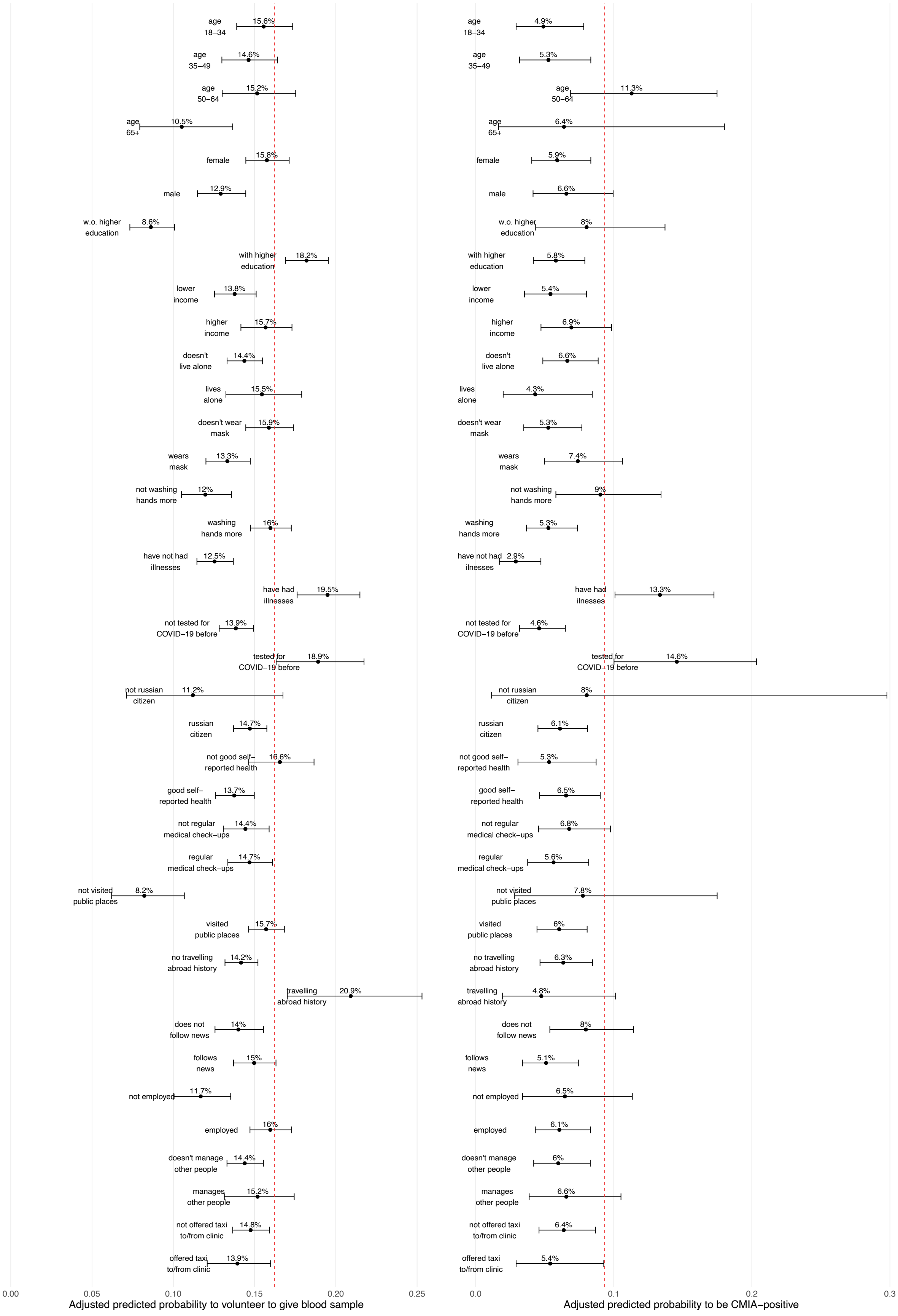

(a) Adjusted predictions to visit clinic test site **(b)** Adjusted predictions to CMIA-seroconvert

This figure reports adjusted predictions from probit model of individual visiting clinic test site (subfigure a) or being CMIA-seropositive (subfigure b) holding all but one regressor at mean levels. Horizontal lines are 95% CI. Dashed vertical red line is unconditional mean propensity.

Figure A2. Adjusted predictions to participate in the study or seroconvert
